# Sacubitril/Valsartan Induces Global Cardiac Reverse Remodeling in Long-Lasting Heart Failure with Reduced Ejection Fraction: Standard and Advanced Echocardiographic Evidences

**DOI:** 10.3390/jcm9040906

**Published:** 2020-03-25

**Authors:** Matteo Castrichini, Paolo Manca, Vincenzo Nuzzi, Giulia Barbati, Antonio De Luca, Renata Korcova, Davide Stolfo, Andrea Di Lenarda, Marco Merlo, Gianfranco Sinagra

**Affiliations:** 1 Division of Cardiology, Cardiothoracovascular Department, Azienda Sanitaria Universitaria Integrata di Trieste, 34149 Trieste, Italy; paolo.manca91@yahoo.it (P.M.); vincenzo_nuzzi@libero.it (V.N.); deluca.antonio.md@gmail.com (A.D.L.); r_korcova@yahoo.it (R.K.); davide.stolfo@gmail.com (D.S.); marco.merlo79@gmail.com (M.M.); gianfranco.sinagra@asuits.sanita.fvg.it (G.S.); 2 Biostatistics Unit, Department of Medical Sciences, University of Trieste, 34149 Trieste, Italy; gbarbati@units.it; 3 S.C. Centro Cardiovscolare, Azienda Sanitaria Universitaria Integrata, 34149 Trieste, Italy; dilenar@units.it

**Keywords:** sacubitril/valsartan, cardiac reverse remodeling, heart failure with reduced ejection fraction, strain

## Abstract

Sacubitril/valsartan reduces mortality in heart failure with reduced ejection fraction (HFrEF) patients, partially due to cardiac reverse remodeling (RR). Little is known about the RR rate in long-lasting HFrEF and the evolution of advanced echocardiographic parameters, despite their known prognostic impact in this setting. We sought to evaluate the rates of left ventricle (LV) and left atrial (LA) RR through standard and advanced echocardiographic imaging in a cohort of HFrEF patients, after the introduction of sacubitril/valsartan. A multi-parametric standard and advanced echocardiographic evaluation was performed at the moment of introduction of sacubitril/valsartan and at 3 to 18 months subsequent follow-up. LVRR was defined as an increase in the LV ejection fraction ≥10 points associated with a decrease ≥10% in indexed LV end-diastolic diameter; LARR was defined as a decrease >15% in the left atrium end-systolic volume. We analyzed 77 patients (65 ± 11 years old, 78% males, 40% ischemic etiology) with 76 (28–165) months since HFrEF diagnosis. After a median follow-up of 9 (interquartile range 6–14) months from the beginning of sacubitril/valsartan, LVRR occurred in 20 patients (26%) and LARR in 33 patients (43%). Moreover, left ventricular global longitudinal strain (LVGLS) improved from −8.3 ± 4% to −12 ± 4.7% (*p* < 0.001), total left atrial emptying fraction (TLAEF) from 28.2 ± 14.4% to 32.6 ± 13.7% (*p* = 0.01) and peak atrial longitudinal strain (PALS) from 10.3 ± 6.9% to 13.7 ± 7.6% (*p* < 0.001). In HFrEF patients, despite a long history of the disease, the introduction of sacubitril/valsartan provides a rapid global (i.e., LV and LA) RR in >25% of cases, both at standard and advanced echocardiographic evaluations.

## 1. Introduction

Cardiac adverse remodeling is associated with poor outcome in patients affected by heart failure with reduced ejection fraction (HFrEF) [1,2]. It is well known that the inhibition of neurohormonal system, through ACE inhibitors (ACE-i), beta-blockers and mineral-corticoid receptor blockers (MRA), improves left ventricular ejection fraction (LVEF) inducing a left ventricular reverse remodeling (LVRR) [3,4,5,6].

Recently, sacubitril/valsartan has been shown to reduce cardiovascular mortality and hospitalizations in HFrEF patients compared to enalapril and also to decrease ventricular arrhythmias and appropriate implantable cardioverter defibrillator (ICD) shocks [7,8]. However, even though one prospective study reported a significant cardiac RR during therapy with sacubitril/valsartan along with a reduction in N-terminal pro B-type natriuretic peptide (NT-proBNP) blood levels [9], data on the precise rates of LVRR and LARR, as defined in literature [2,10], are scarce, mostly in long-lasting diseases. Furthermore, so far, there are few evidences about the evolution of LV and LA advanced echocardiographic parameters under sacubitril/valsartan, despite their prognostic role has recently emerged in the setting of HFrEF patients [11]. Therefore, our study sought to evaluate the rates of global cardiac reverse remodeling, through a multi-parametric standard and advanced echocardiographic evaluation, in a long-lasting HFrEF population.

## 2. Experimental Section

From January 2016 to January 2018 we prospectively enrolled HFrEF patients under optimal medical therapy (OMT) who started sacubitril/valsartan at Heart Failure Clinic of the University Hospital of Trieste. Inclusion criteria were:

1) Diagnosis of symptomatic HFrEF (<40%) despite OMT according to ESC guidelines [12].
2) Availability of a follow-up evaluation at 3 to 18 months including a multi-parametric echocardiographic evaluation. Considering the observational character of our study, the timing of the follow-up evaluation was established by the physician who followed the patient. In case of two echocardiographic evaluations during the follow-up period the last one was considered.
3) Absence of cardiac resynchronization therapy during the study observation and within 12 months before the enrolment (i.e., introduction of sacubitril/valsartan)

The up-titration of the sacubitril/valsartan dosage was performed every two weeks, if tolerated. Changes in doses of other medications were allowed when appropriate. In order to standardize the doses of ACE-i and antagonist of angiotensin receptors (ARB) the following conversion were adopted: Enalapril 40 mg = Ramipril 10 mg, Losartan 100 mg = Ramipril 10 mg. About beta blockers the following conversion were adopted: Carvedilol 50 mg = Bisoprolol 10 mg, Metoprolol 200 mg = Bisoprolol 10 mg.

Echocardiographic studies were performed in left lateral decubitus position using iE33 xMATRIX (Philips Medical Systems, Andover, MA, USA), Vivid E9 or Vivid E95 imaging devices (GE Healthcare, Little Chalfont, UK). Data were digitally stored and analyzed offline using a vendor-independent software (Tomtec-Arena TTA.2, Munich, Germany). All images were acquired at a frame rate of at least 50 frames/sec.

Echocardiographic analysis was performed by operators blinded to other patient’s details.

Left ventricular (LV) dimensions and function were assessed according to the international guidelines [13].

In particular, LV volumes and LVEF were calculated by Simpson’s biplane method. Left atrium (LA) parameters were obtained from an optimized apical four-chamber view, avoiding foreshortening of the atrium. LA volumes were determined using the area-length method. Total LA emptying fraction (TLAEF) was calculated as ((maximal LA volume - minimal LA volume)/maximal LA volume X 100%).

Diastolic function was evaluated according to international guidelines [14]. In particular, mitral E and A-wave velocities were measured with pulsed wave Doppler from the apical 4-chamber view. Tissue Doppler imaging was used to evaluate septal E’. The E/E’ ratio was also derived. Right ventricle fractional area change (RVFAC) and tricuspid annular plane systolic excursion (TAPSE) were used to assess right ventricular (RV) systolic function. Pulmonary artery systolic pressure (PAPs) was estimated by the peak velocity of tricuspid regurgitation using the modified Bernoulli equation.

For speckle tracking analysis, endocardial borders of LV and LA were traced using a chamber-specific tool. End-diastole and end-systole were defined by both ECG and visual assessment of 2D images. The software (Tomtec-Arena TTA.2, Munich, Germany) automatically tracked speckles along the endocardial border of each chamber throughout the cardiac cycle providing the left ventricular global longitudinal strain (LVGLS) and the peak atrial longitudinal strain (PALS). All measurements were obtained from the mean of 3 beats (patients in sinus rhythm) or 5 beats (patients in atrial fibrillation (AF)).

LVRR was defined as an increase in the LVEF ≥ 10% (or LVEF > 50%) associated with a decrease ≥10% in indexed left ventricular end-diastolic diameter (LVEDD) or (LVEDD ≤ 33 mm/m^2^) at follow-up evaluation [2]. LARR was described as a reduction >15% in the left atrium end-systolic volume (LAESV) [10].

### Statistical Analysis

Categorical data are presented as percentages and numbers, normally distributed continuous data as mean ± standard deviation, and non-normally distributed variables as median and interquartile range (IQR). Unpaired and paired Student’s *t*-test was used, when appropriate, for comparison of normally distributed data, while Mann-Whitney test was used for abnormally distributed data. The χ^2^ test or the Fisher test were used, when appropriate, to compare non-continuous variables expressed as proportion. 

Univariable and subsequent possible multivariable logistic regression modelling was performed to assess the effect of the clinical variables LVRR. *p* values are two sided and were considered significant when <0.05.

Intra and Inter observer variability was determined by the ICC (Intraclass Correlation Coefficient) computed respectively on the repeated measurements at 2 different times by 1 experienced reader (M.C.) in 23 randomly selected patients. Then, a second experienced reader (V.N.) performed the analysis in the same 23 patients, providing the interobserver measurements data. This allowed us to achieve 80% power to detect an ICC of 0.80 under the null hypothesis of ICC = 0.50, by using an F-test at a significance level of 0.05 [15].

The agreement between measures for the assessment of LV and LA strain were further explored using Bland-Altman analysis. (Table S1).

All the analyses were performed using IBM Corp. Released 2016. IBM SPSS Statistics for Windows, Version 24.0. Armonk, NY, USA: IBM Corp.

## 3. Results

During the study period a total of 101 patients were enrolled. Among them, 7 patients discontinued the drug due to side effects, 15 were excluded because poor acoustic echocardiographic windows and finally 2 underwent cardiac resynchronization device implantation. Finally, the study population was composed of 77 HFrEF patients with a mean duration of heart failure of 76 (28–165) months. The main characteristics of the patients are described in Table 1. The mean age was 65 ± 11 years, 78% were males, 40% had ischemic heart disease (IHD). Twenty-three percent of patients had New York Heart Association (NYHA) class III. All patients were treated with ACE-i at the highest dose tolerated (mean 5.2 ± 3.2 mg ramipril dose equivalent). Similarly, most of patients were treated with beta-blockers (93.5%; mean 3.2 ± 2 mg/day of bisoprolol dose equivalent) and MRA (60%). Loop diuretics were prescribed to 86% patients.

At baseline, a conspicuous cardiac remodeling was present: mean LVEF was 28 ± 6%, LVEDDi 34 ± 5 mm/m^2^, left ventricular end-diastolic volume indexed (LVEDVi) 101 ± 36 mL/m^2^, left atrial end-systolic volume (LAESV) 110 ± 50 mL. LVGLS and PALS were compromised consistently with the chambers remodelling (−8.3 ± 4% and 10.3 ± 6.9%, respectively).

**Figure 1:**
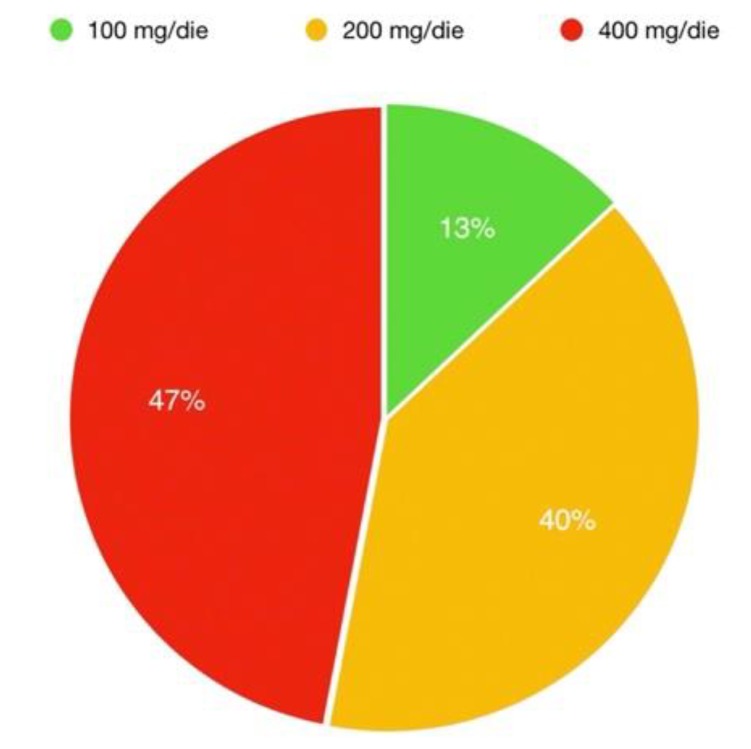
Distribution of doses in population taking sacubitril/valsartan.

After a median follow up of 9 (6–14) months from sacubitril/valsartan introduction, no patient died or experienced any other relevant clinical event. In total, 47% of the population was taking the highest dose of sacubitril/valsartan, 40% the intermediate dose and 13% the lowest dose (Figure 1). No significant differences were observed about the dosages of other medications (Table 1).

At clinical-echocardiographic follow-up evaluation, there was a significant improvement of the mean functional status (NYHA class I from 0% to 21% of the patients, *p* < 0.001). Furthermore, a significant cardiac reverse remodelling emerged: 20 patients (26%) showed a LVRR and 33 (43%) a LARR. 

**Figure 2:**
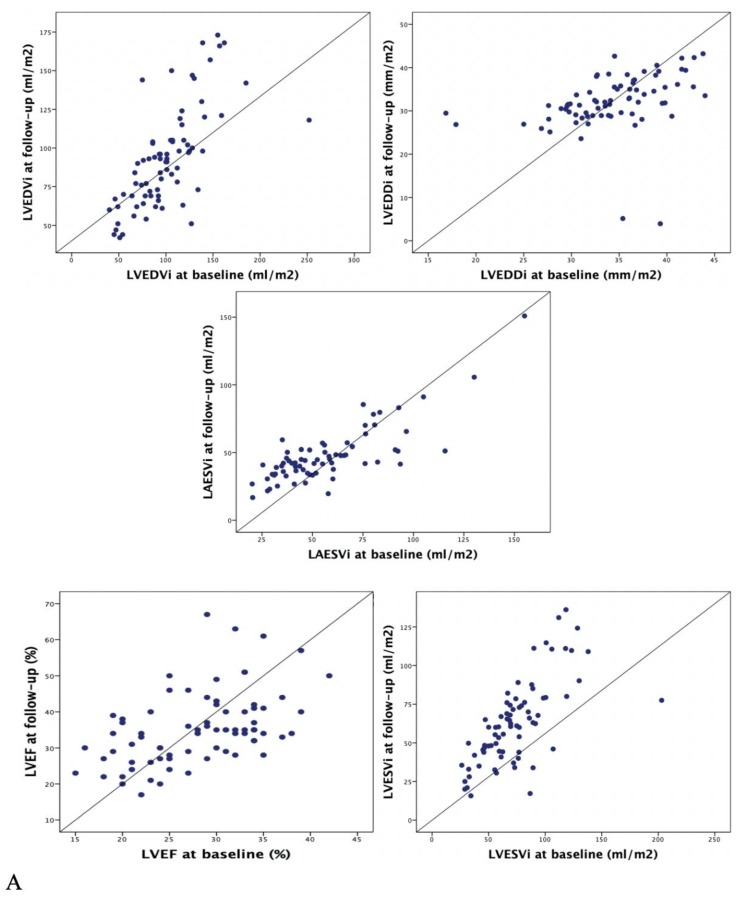

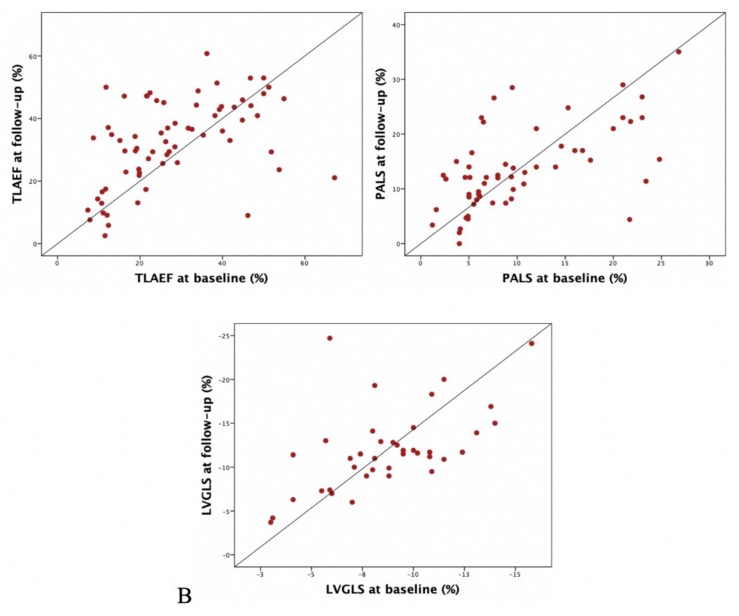
Scatter plots representing conventional (**A**) and advanced (**B**) echocardiographic multiparametric evaluation under sacubitril/valsartan. Note the significant improvement consistent across standard and advanced parameters. LVEDDi, left ventricular end-diastolic diameter indexed; LVEDVi, left ventricular end-diastolic volume indexed; LVEF, left ventricular ejection fraction; LAESV, left atrial end-systolic volume; LVGLS, left ventricular global longitudinal strain; TLAEF, total left atrial emptying fraction; PALS, peak atrial longitudinal strain.

Figure 2 and Table 2 show a significant improvement of all atrial and ventricular parameters considered in echocardiographic evaluation. 

**Figure 3:**
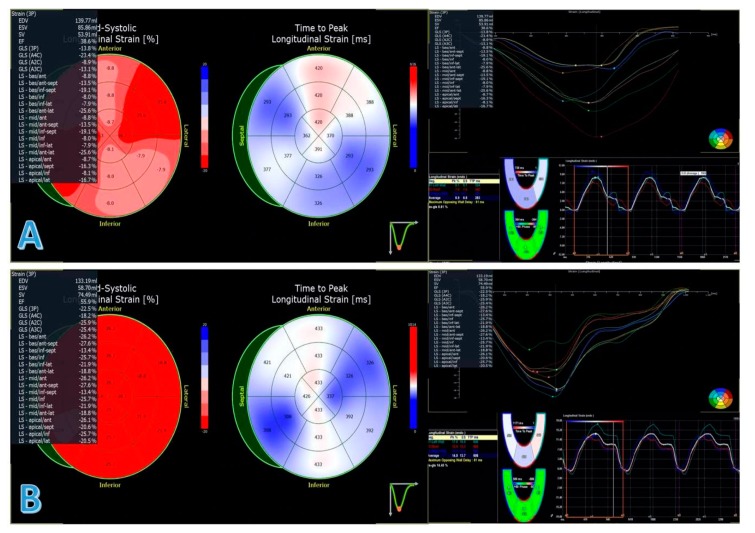
Left ventricular global longitudinal strain and peak atrial longitudinal strain of the same patient at baseline (panel **A**) and at follow-up (panel **B**). EDV, end-diastolic volume; ESV, end-systolic volume; SV, stroke volume; EF, ejection fraction; GLS, global longitudinal strain; LS, longitudinal strain; TLAEF, total left atrial emptying fraction; PALS, peak atrial longitudinal strain; ES-GLS, end-systolic global longitudinal strain.

Concerning traditional echocardiographic findings, mean LVEDDi decreased from 34 ± 5 to 32 ± 7 mm/m^2^ (*p* = 0.006), LVEF increased 28 ± 6% to 35 ± 10% (*p* < 0.001). Regarding LA, LEASVi dropped from 57 ± 26 to 48 ± 21 mL/m^2^ (*p* < 0.001). Concerning advanced echocardiographic parameters, LVGLS improved from −8.3 ± 4% to −12 ± 4.7% (*p* < 0.001). Similarly, TLAEF increased from 28.2 ± 14.2% to 32.6 ± 13.6% (*p* = 0.013) and PALS from −10.3 ± 6.9% to -13.7 ± 7.6% (*p* < 0.001), (Table 3, Figure 3).

At univariable analysis (Table 4), only the shorter duration of the disease was associated with LVRR (51 ± 54 months in patients experiencing LVRR vs. 128 ± 105 months in patients without LVRR, OR 0.988 [C.I. 0.980–0.997] per month; *p* = 0.01). Therefore, a multivariable analysis was not performed.

## 4. Discussion

### Main Findings

Recently, there is an increasing body of data about the effect of Sacubitril/Valsartan on LVRR. In this filed, our analysis adds important elements of novelty, possibly useful in the management of HFrEF patients:

1. This is the first report to consider a comprehensive evaluation of LV and LA using both advanced and standard echocardiographic parameters in this setting, documenting that the improvement of both variables was consistent and conferring value to advanced echocardiography
2. Furthermore, to the best of our knowledge, no previous study reported the proportion of patients reaching LVRR and LARR according to accepted definitions [2,10]
3. Finally, the results provided by our study are of particular interest considering the rapid effect showed by sacubitril/valsartan both on LVRR and on LARR despite the long-lasting diseased patients included

The findings of the Prospective Comparison of Angiotensin Receptor Neprilysin Inhibitor with an Angiotensin Converting Enzyme Inhibitor to Determine Impact on Global Mortality and Morbidity in Heart Failure (PARADIGM HF) trial have been revolutionary for HFrEF management [7]. Indeed, after several years, a novel drug showed a marked reduction in HF mortality and hospitalizations, on top of well consolidated HFrEF treatment [12]. 

Recently, sacubitril/valsartan has been shown to cause a cardiac RR on the top of OMT [9], which could be one of the main determinants of the survival benefit. However, a large meta-analysis demonstrated the capacity of sacubitril/valsartan to induce an average LVEF improvement only of 5.1% [16]. So far, data regarding advanced echocardiography parameters and the proportion of patients on sacubitril/valsartan undergoing a complete LVRR and LARR adopting well defined criteria already published in the literature [2,10], still lack. In fact, despite some clinical reports demonstrated a possible improvement of LVGLS [17] in this setting, they evaluated a very small sample of patients and only a minority (4%) were taking the highest dose of the drug, whilst it has been reported that the amount of LV improvement on sacubitril valsartan might be dose dependent [18].

In this scenario, our experience adds meaningful findings to the current literature. To the best of our knowledge, this is the first study which considers a multi-parametric standard and advanced echocardiographic assessment demonstrating a global cardiac further RR (defined according to validated definitions) induced by sacubitril/valsartan on the top of OMT. Importantly, RR was demonstrated in >25% of patients and the effect of sacubitril/valsartan was quite rapid (i.e., 9 months), despite a mean long-lasting HFrEF under OMT (i.e., >6 years on average). These results claim for a confirmation in larger populations because they raise possible important clinical implications in the management of HFrEF patients, such as a more precise definition of the best timing for switching from ACE-i to sacubitril/valsartan.

We found LVRR in 26% and LARR in 43% of patients, after switching to sacubitril/valsartan. It is important to remark that the positive cardiac RR was likely to be totally due to sacubitril/valsartan, considering that patients undergoing cardiac resynchronization therapy during the study observation were excluded and the remaining HF medications did not significantly change during the follow-up period. Interestingly, it is possible that these results are even underestimated. Indeed, in previous experiences, a LVRR induced by optimal medical therapy has been described from 12 to 24 months of OMT [19], whilst our median follow-up was 9 months. This finding suggests that sacubitril/valsartan could lead to a prompt RR after its introduction. However, a real focused comparison between the rapidity of sacubitril/valsartan and standard OMT in inducing RR is still lacking and it is advocated. Furthermore, an almost double proportion of patients experienced LARR compared to LVRR, underlining the potential impact of sacubitril/valsartan in improving diastolic function in HFrEF patients, as partially emerged from the results of the Efficacy and Safety of LCZ696 Compared to Valsartan, on Morbidity and Mortality in Heart Failure Patients With Preserved Ejection Fraction (PARAGON-HF) trial in heart failure with preserved ejection fraction patients [20].

Concerning advanced echocardiographic evaluation, LVGLS, PALS and TLAEF showed a parallel significant improvement to traditional LV and LA functional parameters. In other settings, LVGLS has been reported to show earlier changes in the LV function compared to LVEF [21]. It might be interesting to document if the LVRR induced by sacubitril/valsartan is anticipated by an earlier improvement in LVGLS to better define the role of speckle tracking analysis in this specific setting.

To the best of our knowledge, the results on PALS and TLAEF are completely new and need further confirmation. However, they should be part of LA assessment, that could be strongly useful for the early detection of RR under therapy in HFrEF patients.

Interestingly, the only parameter negatively associated to the LVRR was a long-standing history of HFrEF (mean 51 months in the LVRR group, 128 months in the non-LVRR group). It appears clear that a long-standing disease already in OMT might be less responsive to switching therapy because of a more consolidated and irreversible adverse cardiac remodeling. Nonetheless, it is noteworthy that patients with a duration of symptoms and OMT longer than 48 months, still exhibited LVRR adding sacubitril/valsartan on top of OMT, highlighting the great potential of the drug.

On the other hand, in our population we did not found any factor associated with LARR after beginning therapy with sacubitril/valsartan (Table S2).

Finally, important considerations on ICD implantation in primary prevention could be raised from the present results. The significant effect of sacubitril/valsartan on all the considered parameters evaluated in this study, also in long-standing diseases, could be consistent with a demonstrated decreased burden of ventricular arrhythmias and ICD appropriate interventions induced by the drug [8,22]. It emerges the need of future prospective trials focused on the cost effectiveness of a further optimization of the therapy shifting to sacubitril/valsartan before ICD implantation.

Some limitations need to be acknowledged. This is not a randomized study, neither we had a matched control population available. We considered a relatively small population, although no previous reports are available on global cardiac RR by standard and advanced echocardiographic evaluation in larger samples. Furthermore, in our analysis follow-up LVEF and GLS evaluations were performed at the same time. In future experiences, it would be definitely important to explore if speckle tracking analysis might allow an earlier detection of LVRR compared to standard echocardiographic evaluation. Finally, we did not have available cardiac magnetic resonance data in our patients while this method can be useful to more precisely characterize the myocardial tissue and to investigate the effect of the drug also on the LV fibrosis. Moreover, sample plasmatic heart failure biomarkers concentrations were not systematically available for many patients.

In conclusion, when applied to daily clinical practice, our study highlights the usefulness of the switching to sacubitril/valsartan in stable HFrEF, despite long-standing diseases. In fact, consistent rates of LARR and LVRR, both at standard and advanced echocardiographic evaluation, could be observed.

## Figures and Tables

**Table 1:** Baseline and follow-up clinical characteristics and medications.

Population *N* = 77	Baseline	Follow-Up	*p* Value
Age (years)	65 ± 11	66 ± 18	N.C.
Male gender, no. %	60 (77.9)	60 (77.9)	N.C
Caucasian race, no. %	77 (100)	77 (100)	N.C.
BMI (Kg/m^2^)	26.9 ± 3.6	27.1 ± 3.7	N.C.
Duration of follow-up (months)	9 (6–14)	N.A.	N.A.
Time since first diagnosis (months)	76 (28–165)	N.A.	N.A.
IHD Etiology, no. %	31 (40.3)	31 (40.3)	N.C
SBP (mmHg)	121 ± 13	118 ± 15	0.088
DBP (mmHg)	73 ± 8	73 ± 8	0.903
HR (b/min)	68 ± 11	67 ± 12	0.416
NYHA Class, no. %			
I	0 (0)	16 (20.8)	
II	59 (76.6)	53 (68.8)	
III	18 (23.4)	8 (10.4)	<**0.001**
IV	0(0)	0(0)	
COPD, no. %	13 (16.9)	13 (16.9)	N.C
Diabetes mellitus, no. %	35 (45.5)	35 (45.5)	N.C
Hypertension, no. %	42 (54.5)	42 (54.5)	N.C
History of AF, no. %	29 (37.7)	29 (37.7)	N.C
Creatinine (mg/dL)	1.2 ± 0.5	1.31 ± 0.49	0.17
Potassium (mmol/L)	4.4 ± 0.4	4.38 ± 0.45	0.99
Beta-blocker, no. %	72 (93.5)	74 (96.1%)	0.71
Beta-blocker bisoprololo dose equivalent (mg)	3.6 ± 2	4 ± 2.7	0.18
ACE-i/ARB, no. %	77 (100)	N.A.	N.A.
ACE-i ramipril dose equivalent (mg)	5.2 ± 3.2	N.A.	N.A.
MRA, no. %	46 (60)	48 (65.8)	0.86
Diuretics, no. %	66 (85.7)	63(81.8)	0.66
Diuretics furosemide dose equivalent dose (mg)	47 ± 59	43 ± 56	0.60
Ivabradine, no. %	10 (13)	7 (9)	0.61
ICD, no. %	53 (68.8)	53 (68.8)	N.C.
CRT, no. %	23 (23.9)	23 (23.9)	N.C.

BMI, body mass indexed; IHD, ischaemic heart disease; SBP systolic blood pressure; DBP, diastolic blood pressure; HR, heart rate; NYHA, New York Heart Association; COPD, chronic obstructive pulmonary disease; DM, diabetes mellitus; AF, atrial fibrillation; ACE-i, angiotensin converting enzyme inhibitors; ARB, angiotensin receptor blockers; MRA mineral corticoid antagonists; ICD, implantable cardioverter defibrillator; CRT, cardiac resynchronization therapy; N.A., not available; N.C., not calculable. In bold *p* value < 0.05. *P* values are estimated by χ^2^ test for categorical variables, i.e: male gender, Caucasian race, IHD etiology, COPD, diabetes mellitus, hypertension, history of AF, beta-blocker no%, ACEi/ARB no%, MRA no%, diuretics no%, ivabradine no%, ICD no%, CRTno%; continuous varables (all the others) are estimated by student’s *t*-test.

**Table 2:** Comparison between baseline and follow-up standard echocardiographic parameters.

Standard Echocardiographic Evaluation	Baseline (*N* = 77)	Folow-up (*N* = 77)	*p* Value
LVEDDi (mm/m^2^)	34 ± 5	32 ± 7	0.006
LVEDVi (mL/m^2^)	101 ± 36	93 ± 32	0.02
LVESVi (mL/m^2^)	74 ± 30	63 ± 27	<0.001
LVEF (%)	28 ± 6	35 ± 10	<0.001
LAESV (mL)	110 ± 50	92 ± 40	<0.001
LAESVi (mL/m^2^)	57 ± 26	48 ± 21	<0.001
RAESA (cm^2^)	18 ± 5.5	17 ± 5	0.011
E/E’	16.7 ± 9	14.8 ± 7	0.11
Restrictive filling pattern no, %	15 (19.5)	10(13)	0.38
MR moderate/severe, *N*, %	25 (32.)	19(24.7)	0.2
PAPs (mmHg)	42 ± 19	38 ± 17	0.122
TAPSE (mm)	19 ± 4	20 ± 5	0.72
RVFAC (%)	42 ± 11	42 ± 10	0.8

LVEDDi, left ventricular end-diastolic diameter indexed; LVEDVi, left ventricular end-diastolic volume indexed; LVESVi, left ventricular end-systolic volume indexed; LVEF, left ventricular ejection fraction; LAESV, left atrial end-systolic volume, LAESVi, left atrial end-systolic volume indexed; LAFAC, left atrial fractional area change; RAESA, right atrial end-systolic area; RAESV right atrial end-systolic volume indexed; MR, mitral regurgitation; PAPs systolic pulmonary artery systolic pressure; TAPSE tricuspid systolic annulus excursion; RVFAC right ventricular fractional shortening area. In bold *p* value < 0.05. *p* values are estimated by χ^2^ test for restrictive filing pattern and MR moderate/severe (categorical variables), all the others (continuous variables) are estimated by student’s *t*-test.

**Table 3:** Comparison between baseline and follow-up advanced echocardiographic parameters.

Advanced Echocardiographic Evaluation	BASELINE (*N* = 77)	Follow-Up (*N* = 77)	*p* Value
LVGLS (%)	−8.3 ± 4	−12 ± 4.7	<0.001
TLAEF (%)	28.2 ± 14.2	32.6 ± 13.6	0.013
PALS (%)	10.3 ± 6.9	13.7 ± 7.6	<0.001

LVGLS, left ventricular global longitudinal strain; TLAEF, total left atrial empting fraction; PALS, peak atrial longitudinal strain. In bold *p* value < 0.05. P values are estimated by student’s *t*-test.

**Table 4:** Univariable analysis for LVRR.

	LVRR (*N* = 20)	No LVRR (*N* = 57)	OR (95% C.I.)	*p* Value
Male sex, no. (%)	14 (70%)	45 (80%)	1.753 (0.549–5.601)	0.343
Age (years)	69 ± 11	64 ± 11	1.038 (0.987–1.091)	0.147
**Duration of the disease (months)**	**28 (9–90)**	**99 (35–221)**	**0.988 (0.980–0.997)**	**0.010**
Follow up (months)	8 (6-11)	10 (6–15)	0.948 (0.859–1.047)	0.291
HR (bpm)	66.8 ± 11	68.3 ± 11	0.987 (0.940–1.035)	0.585
SBP (mmHg)	125 ± 11	119 ± 13	1.038 (0.998–1.080)	0.063
DBP (mmHg)	73 ± 8	72.7 ± 8	1.005 (0.942–1.071)	0.890
COPD, no. (%)	3 (15%)	10 (18%)	0.812 (0.199–3.308)	0.771
Diabetes mellitus, no. (%)	6 (30%)	28 (49%)	0.429 (0.144–1.275)	0.128
Hypertension, no. (%)	10 (50%)	25 (44%)	0.806 (0.290–2.24)	0.680
IHD, no. (%)	5 (25%)	25 (44%)	2.419 (0.773–7.573)	0.129
NYHA Class (average)	2.15 ± 0.366	2.26 ± 0.442	0.504 (0.128–1.984)	0.327
History of AF, no. (%)	8 (40%)	21 (37%)	1.111 (0.391–3.161)	0.843
Creatinine (mg/dL)	1.02 ± 0.43	1.26 ± 0.5	0.197 (0.027–1.440)	0.109
ACE-I/ARB (Ramipril dose equivalent), mg	5.6 ± 3	4.98 ± 3.3	1.054 (0.904–1.228)	0.503
Beta-blockers, no. (%)	19 (95%)	52 (93%)	1.462 (0.154–1.914)	0.741
Beta-blockers (bisoprolol dose equivalent), mg	3.25 ± 2.2	3.64 ± 2.4	0.928 (0.738–1.168)	0.526
Sacubitril/Valsartan full dose, no. (%)	12 (60%)	23 (41%)	2.152 (0.760–6.095)	0.149
MRA, no. (%)	11 (55%)	35 (62%)	0.733 (0.261–2.062)	0.557
Diuretics, no. (%)	17 (85%)	48 (85%)	0.944 (0.224–3.977)	0.938
Diuretics (furosemide dose equivalent), mg	55 ± 28	44 ± 25	1.003 (0.995–1.011)	0.488
Ivabradine, no. (%)	2 (10%)	8 (14%)	0.667 (0.129–3.442)	0.628
CRT, no. (%)	5 (20%)	18 (32%)	0.704 (0.221–2.238)	0.552
LVEF (%)	27 ± 6	28 ± 6	0.979 (0.901–1.063)	0.606
LVEDVi (mL/m^2^)	99 ± 47	101 ± 32	0.998 (0.984–1.013)	0.785
LVESVi (mL/m^2^)	72.5 ± 40	74 ± 26	0.998 (0.981–1.015)	0.808
LAESV (mL)	106 ± 48	111 ± 50	0.998 (0.987–1.009)	0.719
RAESA (cm^2^)	18.4 ± 4.9	18.2 ± 5.4	1.007 (0.911–1.114)	0.890
E/E’	14.6 ± 5.2	17 ± 9.8	0.965 (0.897–1.038)	0.341
Restrictive pattern, no. (%)	3 (14%)	14 (25%)	0.418 (0.083–2.089)	0.288
RVFAC (%)	42 ± 14	38 ± 12	1.023 (0.969–1.080)	0.416
TAPSE (mm)	20 ± 5.8	19 ± 5,9	1.027 (0.925–1.141)	0.612
PAPs (mmHg)	37 ± 15	44 ± 18	0.975 (0.935–1.017)	0.233

HR, heart rate; SBP systolic blood pressure; DBP, diastolic blood pressure; COPD, chronic obstructive pulmonary disease; IHD, ischaemic heart disease; NYHA, New York Heart Association;; AF, atrial fibrillation; ACE-i, angiotensin converting enzyme inhibitors; ARB, angiotensin receptor blockers; MRA mineral corticoid antagonists; CRT, cardiac resynchronization therapy; LVEF, left ventricular ejection fraction; LVEDVi, left ventricular end-diastolic volume indexed; LVESVi, left ventricular end-systolic volume indexed; LAESV, left atrial end-systolic volume; RAESA, right atrial end-systolic area; RVFAC right ventricular fractional area change; TAPSE tricuspid systolic annulus excursion; PAPs pulmonary artery systolic pressure.

## Supplementary Materials

The following are available online at https://www.mdpi.com/2077-0383/9/4/906/s1, Table S1: Intraclass Correlation Coefficients (ICC) results, Table S2: Univariable analysis for LARR.

## References

[1] Burchfield J.S., Xie M., Hill J.A. (2013). Pathological ventricular remodeling: Mechanisms: Part 1 of 2. Circulation.

[2] Merlo M., Pyxaras S.A., Pinamonti B., Barbati G., Di Lenarda A., Sinagra G. (2011). Prevalence and prognostic significance of left ventricular reverse remodeling in dilated cardiomyopathy receiving tailored medical treatment. J. Am. Coll. Cardiol..

[3] Matsumori A. (2003). Assessment of Response to Candesartan in Heart Failure in Japan (ARCH-J) Study Investigators. Efficacy and safety of oral candesartan cilexetil in patients with congestive heart failure. Eur. J. Heart Fail.

[4] Cicoira M., Zanolla L., Rossi A., Golia G., Franceschini L., Brighetti G., Marino P., Zardini P. (2002). Long-term, dose-dependent effects of spironolactone on left ventricular function and exercise tolerance in patients with chronic heart failure. J. Am. Coll. Cardiol.

[5] Greenberg B., Quinones M.A., Koilpillai C., Limacher M., Shindler D., Benedict C., Shelton B. (1995). Effects of long-term enalapril therapy on cardiac structure and function in patients with left ventricular dysfunction. Results of the SOLVD echocardiography substudy. Circulation.

[6] Groenning B.A., Nilsson J.C., Sondergaard L., Fritz-Hansen T., Larsson H.B., Hildebrandt P.R. (2000). Antiremodeling effects on the left ventricle during beta-blockade with metoprolol in the treatment of chronic heart failure. J. Am. Coll. Cardiol..

[7] McMurray J.J.V., Packer M., Desai A.S., Gong J., Lefkowitz M.P., Rizkala A.R., Rouleau J.L., Shi V.C., Solomon S.D., Swedberg K. (2014). Angiotensin-neprilysin inhibition versus enalapril in heart failure. N. Engl. J. Med..

[8] Diego C., Gonzalez-Torres L., Nunez J.M., Centurion I.R., Martin-Langerwerf D.A., Sangio A.D., Chochowski P., Casasnovas P., Blazquez J.C., Almendral J. (2018). Effects of angiotensin-neprilysin inhibition compared to angiotensin inhibition on ventricular arrhythmias in reduced ejection fraction patients under continuous remote monitoring of implantable defibrillator devices. Heart Rhythm.

[9] Januzzi J.L., Prescott M.F., Butler J., Felker G.M., Maisel A.S., McCague K., Camacho A., Piña I.L., Rocha R.A., Shah A.M. (2019). PROVE-HF Investigators. Association of Change in N-Terminal Pro-B-Type Natriuretic Peptide Following Initiation of Sacubitril-Valsartan Treatment With Cardiac Structure and Function in Patients With Heart Failure With Reduced Ejection Fraction. JAMA.

[10] Thomas L., Abhayaratna W.P. (2017). Left Atrial Reverse Remodeling, Mechanisms, Evaluation, and Clinical Significance. J. Am. Coll. Cardiol.

[11] Bijl P.V.D., Kostyukevich M.V., Khidir M., Marsan A.N., Delgado V., Bax J.J. (2019). Left ventricular remodelling and change in left ventricular global longitudinal strain after cardiac resynchronization therapy: Prognostic implications. Eur. Heart. J. Cardiovasc. Imag..

[12] Ponikowski P., Voors A.A., Anker S.D., Bueno H., Cleland J.G., Coats A.J., Falk V., González-Juanatey J.R., Harjola V.P., Jankowska E.A. (2016). Guidelines for the diagnosis and treatment of acute and chronic heart failure: The Task Force for the diagnosis and treatment of acute and chronic heart failure of the European Society of Cardiology (ESC). Developed with the special contribution of the Heart Failure Association (HFA) of the ESC. Eur. Heart J..

[13] Lang R.M., Badano L.P., Mor-Avi V., Afilalo J., Armstrong A., Ernande L., Flachskampf F.A., Foster E., Goldstein S.A., Kuznetsova T. (2015). Recommendations for cardiac chamber quantification by echocardiography in adults: An update from the American society of echocardiography and the European association of cardiovascular imaging. Eur. Heart. J. Cardiovasc. Imag..

[14] Nagueh S.F., Smiseth O.A., Appleton C.P., Byrd B.F., Dokainish H., Edvardsen T., Flachskampf F.A., Gillebert T.C., Klein A.L., Lancellotti P. (2016). Recommendations for the Evaluation of Left Ventricular Diastolic Function by Echocardiography: An Update from the American Society of Echocardiography and the European Association of Cardiovascular Imaging. J. Am. Soc. Echocardiogr..

[15] Walter S.D., Eliasziw M., Donner A. (1998). Sample size and optimal designs for reliability studies. Stat. Med..

[16] Aimo A., Emdin M., Maisel A.S. (2019). Sacubitril/Valsartan, Cardiac Fibrosis, and Remodeling in Heart Failure. J. Am. Coll. Cardiol..

[17] Pandey A.C., Pelter M., Montgomery P., Kuo R., Shen C., Sidhu R., Lerner D., Billick K., Hay B., Loveday A. (2019). Changes in Ejection Fraction And Global Longitudinal Strain Assessment In Patients With Heart Failure With Reduced Ejection Fraction After Therapy With Sacubitril/Valsartan. J. Am. Coll. Cardiol..

[18] Martens P., Beliën H., Dupont M., Vandervoort P., Mullens W. (2018). The reverse remodeling response to sacubitril/valsartan therapy in heart failure with reduced ejection fraction. Cardiovasc. Ther..

[19] Merlo M., Caiffa T., Gobbo M., Adamo L., Sinagra G. (2018). Reverse remodeling in Dilated Cardiomyopathy: Insights and future perspectives. Int. J. Cardiol. Heart Vasc..

[20] Solomon S.D., McMurray J.J.V., Anand I.S., Ge J., Lam C.S.P., Maggioni A.P., Martinez F., Packer M., Pfeffer M.A., Pieske B. (2019). PARAGON-HF Investigators and Committees. Angiotensin-neprilysin inhibition in heart failure with preserved ejection fraction. N. Engl. J. Med.

[21] Solomon S.D., McMurray J.J.V., Anand I.S., Ge J., Lam C.S.P., Maggioni A.P., Martinez F., Packer M., Pfeffer M.A., Pieske B. (2017). Early recovery of left ventricular function after revascularization of coronary artery disease detected by myocardial strain. Biomed. Res..

[22] Martens P., Nuyens D., Rivero-Ayerza M., Van Herendael H., Vercammen J., Ceyssens W., Luwel E., Dupont M., Mullens W. (2019). Sacubitril/valsartan reduces ventricular arrhythmias in parallel with left ventricular reverse remodeling in heart failure with reduced ejection fraction. Clin. Res. Cardiol..

