# Supplementary material for: Sacubitril/Valsartan Induces Global Cardiac Reverse Remodeling in Long-Lasting Heart Failure with Reduced Ejection Fraction: Standard and Advanced Echocardiographic Evidences
Source: J Clin Med. 2020 Mar 25;9(4):906. doi: 10.3390/jcm9040906 (PMC7230879; doi:10.3390/jcm9040906)
Supplement: Supplementary file 1 [file jcm-09-00906-s001.pdf]

Supplementary Materials

Supplementary Table S1. Intraclass Correlation Coefficients (ICC) results.

	Intra-Observer ICC (95% CI)	Inter-Observer ICC (95% CI)
LVGLS (%)	0.74 (0.51–0.89)	0.92 (0.81–0.95)
PALS (%)	0.98 (0.97–0.99)	0.98 (0.96–0.99)

LVGLS, left ventricular longitudinal strain; PALS, peak atrial longitudinal strain.

Supplementary Table S2. Univariable analysis for LARR.

	LARR (*N* = 33)	No LARR (*N* = 37)	OR (95% C.I.)	*p* value
Male sex, no. (%)	8 (24.2 %)	8 (21.6%)	1.16 (0.380–3.543)	0.794
Age (years)	68 ± 11	64 ± 13	1.0380(0.986–1.075)	0.185
Duration of the disease (months)	95 (13 – 192)	136(39 – 194)	1.002 (0.997–1.007)	0.360
Follow up (months)	9.3 ± 5.5	10.8 ± 5.6	0.971 (0.894–1.054)	0.971
HR (bpm)	67.7 ± 11	68.6 ± 11	0.987 (0.940–1.035)	0.585
SBP (mmHg)	120 ± 12	121 ± 15	0.995 (0.961–1.030)	0.775
DBP (mmHg)	71 ± 8	74 ± 7	0.955(0.894–1.019)	0.955
COPD, no. (%)	8 (24.2%)	4 (10.8%)	2.640 (0.714–9.764)	0.146
Diabetes mellitus, no. (%)	17 (51.5%)	14 (37.8 %)	1.746 (0.673–4.525)	0.317
Hypertension, no. (%)	20 (60.6%)	18 (48.6%)	1.624 (0.628–4.200)	0.317
IHD, no. (%)	19 (57.6)	24 (64.9%)	0.735 (0.280–1.077)	0.532
NYHA Class (average)	2.2 ± 0.41	2.3 ± 0.46	0.636 (0.213–1.897)	0.417
History of AF, no. (%)	10 (30.3%)	15 (40.5 %)	1.111 (0.391–3.161)	0.843
Creatinine (mg/dL)	1.25 ± 0.61	1.23 ± 0.4	0.923 (0.317–2.688)	0.883
BNP (pg/mL)	262 (116-679)	231(133–546)	1.000 (0.998–1.003)	0.779
ACE-I/ARB (Ramipril dose equivalent), mg	4.9 ± 2.1	6 ± 3.9	1.124 (0.905–1.286)	0.091
Beta-blockers, no. (%)	30 (90.9%)	35 (94.6%)	0.571 (0.089–3.650)	0.554
Beta-blockers (bisoprolol dose equivalent), mg	3.7 ± 2.5	3.4 ± 2.4	0.571 (0.089–3.650)	0.554
Sacubitril/Valsartan full dose, no. (%)	18 (54.5%)	15 (40.5%)	1.21 (0.962–1.42)	0.07
MRA, no. (%)	24 (64.9%)	20 (60.6%)	0.833 (0.316–2.201)	0.713
Diuretics, no. (%)	28 (84.8%)	32 (86.5%)	0.875 (0.229–3.339)	0.875
Diuretics (furosemide dose equivalent), mg	49 ± 54	50 ± 71	1.000 (0.993–1.008)	0.928
Ivabradine, no. (%)	4 (12.1%)	6 (16.2%)	0.713 (0.182–2.784)	0.626
CRT, no. (%)	13 (39.4%)	10 (27%)	1.755 (0.641–4.804)	0.274
LVEF (%)	28 ± 5	28 ± 6	0.985 (0.913–1.063)	0.697
LVEDVi (mL/m^2)	102 ± 40	102 ± 32	1.000 (0.987–1.013)	0.973
LVESVi (mL/m^2)	74 ± 34	75 ± 38	0.999 (0.984–1.015)	0.915
LAESA (cm^2)	29 ± 7	27 ± 9	0.976 (0.912–1.045)	0.260
LAESV (mL)	121 ± 46	100 ± 52	1.009 (0.999–1.019)	0.089
RAESA (cm^2)	19 ± 5.1	18 ± 5.8	1.049 (0.959–1.149)	0.296
E/E′	17.5 ± 11	15.3 ± 7	1.029 (0.970–1.092)	0.346
Restrictive pattern, no. (%)	6(19%)	7(21,9%)	0.857 (0.252–2.913)	0.805
RVFAC (%)	41 ± 10	39 ± 13	1.009 (0.959–1.061)	0.732
TAPSE (mm)	21 ± 6	19 ± 4.2	1.075 (0.951–1.216)	0.249
PAPs (mmHg)	45 ± 18	38 ± 17	1.022 (0.986–1.060)	0.239

HR, heart rate; SBP systolic blood pressure; DBP, diastolic blood pressure; COPD, chronic obstructive pulmonary disease; IHD, ischaemic heart disease; NYHA, New York Heart Association;; AF, atrial fibrillation; BNP, B-type natriuretic peptide; ACE-i, angiotensin converting enzyme inhibitors; ARB, angiotensin receptor blockers; MRA mineral corticoid antagonists; CRT, cardiac resynchronization therapy; LVEF, left ventricular ejection fraction; LVEDVi, left ventricular end-diastolic volume indexed; LVESVi, left ventricular end-systolic volume indexed; LAESA, left atrial end-systolic area;

LAESV, left atrial end-systolic volume; RAESA, right atrial end-systolic area; RVFAC right ventricular fractional area change; TAPSE tricuspid systolic annulus excursion; PAPs systolic pulmonary artery systolic pressure.
